# Health researchers’ experiences, perceptions and barriers related to sharing study results with participants

**DOI:** 10.1186/s12961-019-0422-5

**Published:** 2019-03-04

**Authors:** Christopher R. Long, Rachel S. Purvis, Elizabeth Flood-Grady, Kim S. Kimminau, Robert L. Rhyne, Mark R. Burge, M. Kathryn Stewart, Amy J. Jenkins, Laura P. James, Pearl A. McElfish

**Affiliations:** 1 College of Medicine, University of Arkansas for Medical Sciences Northwest, 1125 N. College Ave, Fayetteville, AR 72703 United States of America; 2 Office of Community Health and Research, University of Arkansas for Medical Sciences Northwest, 1125 N. College Ave, Fayetteville, AR 72703 United States of America; 3 STEM Translational Communication Center, College of Journalism and Communications and Recruitment Center, Clinical Translational Science Institute, University of Florida, 1185 Stadium Road, Gainesville, FL 32611 United States of America; 4 Department of Family Medicine, University of Kansas Medical Center, 3901 Rainbow Blvd, Kansas City, KS 66160 United States of America; 5 Department of Family and Community Medicine, School of Medicine, University of New Mexico, MSC09 5040, Albuquerque, NM 87131 United States of America; 6 Clinical and Translational Science Center, Health Science Center, University of New Mexico, Albuquerque, NM 87131 United States of America; 7 Fay W. Boozman College of Public Health, University of Arkansas for Medical Sciences, 4301 W. Markham, Little Rock, AR 72205 United States of America; 8 Translational Research Institute, University of Arkansas for Medical Sciences, 4301 W. Markham, Little Rock, AR 72205 United States of America; 9 Department of Pediatrics, University of Arkansas for Medical Sciences, 4301 W. Markham, Little Rock, AR 72205 United States of America

**Keywords:** Research dissemination, dissemination survey, results sharing, research results, health research dissemination, research communication

## Abstract

**Background:**

Although research participants are generally interested in receiving results from studies in which they participate, health researchers rarely communicate study findings to participants. The present study was designed to provide opportunity for a broad group of health researchers to describe their experiences and concerns related to sharing results (i.e. aggregate study findings) with research participants.

**Methods:**

We used a mixed–methods concurrent triangulation design, relying on an online survey to capture health researchers’ experiences, perceptions and barriers related to sharing study results with participants. Respondents were health researchers who conduct research that includes the consent of human subjects and hold a current appointment at an accredited academic medical institution within the United States. For quantitative data, the analytic strategy focused on item-level descriptive analyses. For the qualitative data, analyses focused on a priori themes and emergent subthemes.

**Results:**

Respondents were 414 researchers from 44 academic medical institutions; 64.5% reported that results should always be shared with participants, yet 60.8% of respondents could identify studies in which they had a leadership role where results were not shared. Emergent subthemes from researchers’ reasons why results should be shared included participant ownership of findings and benefits of results sharing to science. Reasons for not sharing included concerns related to participants’ health literacy and participants’ lack of desire for results. Across all respondents who described barriers to results sharing, the majority described logistical barriers.

**Conclusions:**

Study findings contribute to the literature by documenting researchers’ perspectives and experiences about sharing results with research participants, which can inform efforts to improve results sharing. Most respondents indicated that health research results should always be shared with participants, although the extent to which many respondents described barriers to results sharing as well as reported reasons not to share results suggests difficulties with a one-size-fits-all approach to improving results sharing.

## Background

Health research participants are generally interested in receiving the results of the studies in which they participate [1–7]. Certain funding agencies, such as the Patient-Centered Outcomes Research Institute and Agency for Healthcare Research and Quality, also emphasise the importance of disseminating research results to non-academic audiences, which can include participants and their communities [8–10]. Nevertheless, despite participant interest in receiving study results, research results are seldom communicated to participants, including individuals who participate in community-based participatory research (CBPR) [6, 11]. Although researchers express support for sharing scientific results with research participants [11–16] and believe participants are interested in receiving study results [14], evidence suggests that researchers rarely share results with participants [11, 14, 15].

Few studies have explored researchers’ experiences, attitudes and barriers related to sharing study results with participants (i.e. sharing de-identified, aggregate information about study findings and/or study progress updates to study participants, through means other than publication of peer-reviewed manuscripts), which has left a significant gap in the literature. The few published studies have focused on the perspectives of cancer [14] or haematology [15] researchers and show that researchers are interested in sharing results but have not typically done so and/or do not currently have a plan for results sharing. The existing literature suggests that sharing results with participants is hampered by pragmatic questions concerning how to do so [17]. Until now, there has been little opportunity for broad groups of health researchers to provide detailed accounts of their experiences and concerns related to sharing results with research participants. The present study addresses that gap in knowledge by characterising – both quantitatively and in the researchers’ own words – health researchers’ attitudes, experiences and barriers related to sharing results with participants. The present study focuses on health researchers in the United States, where, in contrast to other countries (e.g. Australia [18] and the United Kingdom [19, 20]), it is not necessarily normative for health research ethics review boards to encourage – or in some cases require – researchers to share aggregate study findings with participants.

## Methods

A mixed-methods concurrent triangulation design was used. Studies using this design collect qualitative and quantitative data in one simultaneous phase, so that both qualitative and quantitative data can be used to address the research question [21–27]. For this study, the investigative team developed a mixed-methods survey to assess health researchers’ experiences, perceptions and barriers related to sharing study results with participants. Survey development and study recruitment were implemented by a team of investigators from four universities with Clinical and Translational Science Award (CTSA) programme sites. The CTSA programme, funded by the National Institutes of Health’s National Center for Advancing Translational Sciences, is a national network of over 50 medical research centres that collaborate on ways to improve the translational research process and increase patient access to innovative healthcare [28]. The investigative team developed the survey items, incorporating four rounds of item refinement before a final survey draft was approved via consensus. Throughout the survey, the investigative team defined sharing results with participants as the returning of de-identified, aggregate information about study findings and/or study progress updates to study participants, through means other than publication of peer-reviewed manuscripts. A detailed description of the study protocol is published elsewhere [29].

### Participants and recruitment

Inclusion criteria stipulated that participants were aged 18 and older, had a current faculty appointment or a postdoctoral appointment at an accredited academic medical institution in the United States, and were health researchers who conduct research that includes consent of human subjects. Participants were primarily recruited through universities with CTSAs and through universities with Centers for Disease Control and Prevention-funded Prevention Research Centers (PRCs) [30]. To recruit researchers, an email notification was sent to the Principal Investigators (PIs) at CTSAs and PRCs. This notification provided a brief overview of the study and asked CTSA and PRC PIs to assist in notifying health researchers at their institutions about the opportunity to participate in an online survey about the dissemination of research results. The investigative team provided the PIs with a survey invitation template to use to invite their institution’s investigators to participate in the survey. The invitation template described the survey as a “*survey about your experiences with returning study results to participants in health research*.” The invitation template also listed the survey inclusion criteria and a link to the online survey. Additionally, some researchers who completed the survey forwarded the survey invitation to other health researchers.

All participants who met the inclusion criteria were included in the study. After clicking on the link to the online survey, participants provided consent electronically, confirmed their eligibility according to the inclusion criteria, and were then directed to participate. Respondents were not compensated for completing the survey. Two weeks after sending the first email, the research team sent a second email asking PIs to send a reminder invitation to their investigators. In August 2018, upon completion of data analyses for this study, the team returned a summary of the survey’s results via email to all respondents who requested the results. This study was determined to be exempt by the institutional review board (IRB) at the University of Arkansas for Medical Sciences (#205983).

### Survey

The survey was administered online via REDCap [31]. In pilot testing, most respondents completed the survey in fewer than 10 minutes. The survey used Likert-type scales, multiple-response items, yes/no items, and sliders to capture experiences and opinions related to sharing study results with health research participants. The survey was divided into four sections, capturing respondents’ (1) general opinion of returning results to participants in health research studies, (2) experiences in a specific recent study in which they did not share results with participants, (3) perceived barriers to sharing results with participants, and (4) demographic characteristics and professional background. Barriers assessed by the survey included financial barriers, ethical concerns, logistical/methodological/skill-related barriers, and systems barriers (e.g. lack of professional incentive to share results with participants). Throughout the survey, eight open-ended items encouraged respondents to describe experiences, list examples or explain their rationale for responses after closed response questions. These open-ended questions allowed respondents to provide in-depth explanations in their own words about their experiences, perceptions and barriers related to sharing results with research participants.

### Analytic strategy

For the Likert-type scales, multiple-response items, yes/no items and sliders, the analytic strategy focused on presenting results of item-level descriptive analyses, including proportions, counts, or means and standard deviations. Prior to completing the qualitative analysis, all participants were assigned a unique participant identification number (e.g. #245). For the qualitative data, a coding template was identified as the most appropriate strategy to analyse the large number of open-end responses that were collected [32, 33]. The investigative team developed a preliminary coding template with three broad a priori themes from the survey. A priori themes included (1) why researchers should share results, (2) why researchers should not share results, and (3) barriers to sharing research results. Three experienced qualitative researchers independently coded the data for a priori themes and emergent subthemes. Investigators developed a detailed codebook and coding template. The emergent codes were identified, discussed, and incorporated into a detailed codebook and coding template. Comprehensive coding of all qualitative data followed using the codebook and coding template. Two additional researchers provided critical review of the coded data, ensuring analytic rigor and reliability by confirming that the data and illustrative excerpts were extracted to the correct domain. Discussion of discrepancies in data interpretation led to resolving differences via consensus among the coders.

Given the large numbers of participants and subthemes, the goal of this article is to provide an overview of emergent themes. Subsequent articles will provide greater detail on specific thematic areas. After experiences and perceptions were collected and organised into thematic categories, illustrative quotes that best represented the emergent themes were selected. To provide overview information about the relative frequencies with which each subtheme was mentioned by respondents, the coded data were used to calculate the percentage of respondents who mentioned each subtheme in their open-ended responses [34].

## Results

### Respondent characteristics

Between March 8 and April 26, 2017, 414 respondents consented electronically and responded to at least one survey item, and 355 respondents completed the survey. Respondents listed 44 different academic institutions in response to a survey item asking them to identify the universities at which their current primary appointment was held.

The respondents’ demographic characteristics are presented in Table 1. The majority of respondents were female (57.7%) and respondents’ mean age was 50.6 years (*SD* = 11.33). Across all respondents, 56.4% held a PhD and 65.2% had their primary academic appointment in Medicine. Respondents reported completing a median of seven health research studies as PI, Co-PI, or Co-Investigator (Co-I) (range 0 to 200); 73% of respondents reported having served in one of those roles for projects funded by the National Institutes of Health, which was the highest proportion for any funding agency we assessed. When asked how much of their full-time equivalent (FTE) effort was devoted to research, respondents’ median response was 50.0% of their FTE, with 40.1% of respondents reporting spending over 66.7% of their FTE on research. While 52.6% of researchers indicated that none of their research could be considered CBPR, 16.7% reported that over one-third of their research could be considered CBPR.

**Table 1:** Characteristics of survey respondents

	Number (% of survey respondents) or Mean ± SD
Gender (*n* = 350)	
Female	202 (57.7%)
Male	147 (42.0%)
Other	1 (0.3%)
Age (*n* = 311)	50.6 ± 11.3
Degrees held (*n* = 353)^a^	
PhD	199 (56.4%)
MD	158 (44.8%)
MPH	38 (10.8%)
MA/MSc as highest degree	5 (1.4%)
PharmD	2 (0.6%)
Other	21 (5.9%)
Primary academic appointment (*n* = 359)	
Medicine	234 (65.2%)
Public health	30 (8.4%)
Allied health professions	24 (6.7%)
Nursing	21 (5.8%)
Optometry	5 (1.4%)
Pharmacy	5 (1.4%)
Psychology	5 (1.4%)
Other	35 (9.7%)
Ever served as PI, Co-PI or Co-I for the following funders (*n* = 359)^a^	
NIH	262 (73.0%)
CDC	53 (14.8%)
AHRQ	42 (11.7%)
PCORI	39 (10.9%)
Other	186 (51.8%)
Number of completed health research studies as PI, Co-PI or Co-I (*n* = 358)	14.0 ± 22.6
Percent FTE allocated to research (*n* = 356)	52.3 ± 29.8
0.0% FTE	2 (0.6%)
1.0%–33.3% FTE	107 (30.0%)
33.4%–66.7% FTE	105 (29.4%)
66.8%–99.9% FTE	123 (34.5%)
100% FTE	20 (5.6%)
Percent of research defined as CBPR (*n* = 353)	15.7 ± 27.2
0.0% of research as CBPR	185 (52.6%)
1.0%–33.3% of research as CBPR	108 (30.7%)
33.4%–66.7% of research as CBPR	25 (7.1%)
66.8%–99.9% of research as CBPR	24 (6.8%)
100% FTE	10 (2.8%)

Note. Percentages are based on the number of valid responses for each item

^a^Respondents could select more than one response

*AHRQ* Agency for Healthcare Research and Quality, *CBPR* Community-based participatory research, *CDC* Centers for Disease Control and Prevention, *Co-I* co-investigator, *Co-PI* co-principal investigator, *FTE* full-time equivalent, *NIH* National Institutes of Health, *PCORI* Patient-Centered Outcomes Research Institute, *PI* principal investigator

### Responses to quantitative and categorical items

#### Beliefs and experiences related to results sharing with participants

Table 2 presents descriptive statistics on respondents’ beliefs and experiences of sharing results with participants. In response to a question asking whether respondents believe health research results should always be shared with study participants, 64.5% responded affirmatively while 15.7% responded negatively. The remaining 19.8% stated they were not sure.

**Table 2:** Respondents’ general beliefs and specific experiences related to sharing results

	Number (% of survey respondents) or Mean ± SD
Do you think the results of health research studies should always be shared with participants involved in the studies? (*n* = 414)	
Yes	267 (64.5%)
Not sure	82 (19.8%)
No	65 (15.7%)
Can you identify a study that meets the following criteria: (1) You were principal investigator or co-investigator with decision-making power for the study; (2) data analyses were completed at least 1 year ago; (3) the study involved human participants who gave informed consent (or biological material from humans who gave informed consent); and (4) you did not share results with the study’s participants? (*n* = 408)	
Yes	232 (56.9%)
No	176 (43.1%)
At any point during the life of the study you identified, had you considered returning results to participants (not including academic publications/presentations)? (*n* = 148)^a^	
I had not considered returning results to participants	65 (43.9%)
I had an intention to return results but no specific plan to do so	46 (31.1%)
I had considered returning results but decided not to do it	25 (16.9%)
I had an intention to return results and a specific plan to do so	12 (8.1%)
At any point, did you tell the participants in the study you identified that you would share the aggregated results of this study with them? (For example, some researchers include such language in the informed consent process) (*n* = 147)	
No	104 (70.7%)
Unsure	23 (15.6%)
Yes	20 (13.6%)
Which of the following are ways in which the results of the study you identified were disseminated? (*n* = 144)^b^	
Academic publication	132 (91.7%)
Academic conferences	120 (83.3%)
Verbal (community meetings) summary to the community/general public about the results of the study	36 (25.0%)
Press release or other mass media (e.g. radio, news website, newspaper, etc.)	31 (21.5%)
Written summary to the community/general public about the results of the study	26 (18.1%)
Other ways	9 (6.3%)
Policy briefs	6 (4.2%)
I/we made no effort to disseminate the study’s results	5 (3.5%)

Note. Percentages are based on the number of valid responses for each item

^a^Up to 84 respondents who were supposed to be routed to this item and the two items below were not (due to an error in survey flow programming that was corrected for subsequent respondents)

^b^Respondents could select more than one response

Respondents were asked to answer questions about a specific study from their past that met the following criteria: (1) the respondent was PI or Co-I with decision-making power for the study, (2) the study involved human participants who provided informed consent, (3) data analyses were completed at least 1 year ago, and (4) results were not shared with participants. More than half (56.9%) of respondents could identify a specific study that met these criteria. In 60.8% of the specific studies where results were not shared with participants, respondents had either not considered sharing results with participants (43.9%), or they had considered sharing results with participants but decided against it (16.9%). In 31.1% of the specific studies where results were not shared with participants, respondents reported that they had an intention to share results with participants, but no specific plan for how to do so. In 8.1% of the specific studies where results were not shared with participants, respondents reported both an intention to share results with participants as well as a specific plan for doing so.

Among the specific studies where results were not shared with participants, 91.7% resulted in academic publication, 83.3% were presented at academic conferences, 25.0% were presented at community meetings, and 21.5% were presented via press release and/or other mass media. Among the specific studies where results were not shared with participants, 13.6% of respondents had told participants that they would receive results. Among those 13.6% of studies in which participants were told that they would receive results, 70.0% were studies in which researchers reported an intention to return results.

#### Barriers to results sharing

Respondents were asked to use sliders to indicate the proportion of their health research studies in which four specific factors served as a barrier to sharing results with research participants. For these four items, the response scale ranged from 0% (not at all a barrier) to 100% (always a barrier). Table 3 presents a descriptive summary of these responses. Financial barriers (e.g. lack of money to fund efforts to share results with participants), systems barriers (e.g. lack of career-related incentives to share results with participants), logistical/methodological/skill-related barriers (e.g. lack of knowledge about how to disseminate results to lay audiences), and ethical concerns (e.g. concerns about how participants will understand or use the results) were each considered by over 80% of respondents to be a barrier for at least some of their studies. Respondents’ specific experiences related to each of these barriers are described in greater detail below.

**Table 3:** Respondents’ perceptions of the prevalence of specific barriers to sharing results with participants in their studies

	Mean ± SD or Number (% of survey respondents)
For what proportion of your studies do you believe each of the following has been a barrier to sharing your results with research participants? (*n* = 376)	
Financial barriers (*n* = 378)	48.5% ± 36.0
0.0% – Not at all a barrier	52 (13.8%)
1.0%–33.3% of my studies	105 (27.8%)
33.4%–66.7% of my studies	73 (19.3%)
66.8%–99.9% of my studies	114 (27.5%)
100% – Always a barrier	34 (9.0%)
Logistical/methodological/skill-related barriers (*n* = 378)	47.7% ± 34.1
0.0% – Not at all a barrier	43 (11.4%)
1.0%–33.3% of my studies	105 (27.9%)
33.4%–66.7% of my studies	87 (23.1%)
66.8%–99.9% of my studies	110 (29.3%)
100% – Always a barrier	31 (8.2%)
Systems barriers (*n* = 376)	45.3% ± 35.1
0.0% – Not at all a barrier	67 (17.8%)
1.0%–33.3% of my studies	85 (22.6%)
33.4%–66.7% of my studies	102 (27.1%)
66.8%–99.9% of my studies	96 (25.5%)
100% – Always a barrier	26 (6.9%)
Ethical barriers (*n* = 376)	38.5% ± 30.7
0.0% – Not at all a barrier	60 (15.9%)
1.0%–33.3% of my studies	126 (33.3%)
33.4%–66.7% of my studies	98 (25.9%)
66.8%–99.9% of my studies	85 (22.5%)
100% – Always a barrier	9 (2.2%)

Note. Percentages are based on the number of valid responses for each item

### Responses to open-ended items

From the three a priori themes (‘Why researchers should share results’, ‘Why researchers should not share results’, and ‘Barriers researchers encounter’), several subthemes emerged (Table 4). For each a priori theme and emergent subthemes, the most salient findings based on analyses of the collective responses are presented below, as well as the percentage of respondents who mentioned each specific subtheme.

**Table 4:** Emergent subthemes (and percentage of survey respondents who mentioned each subtheme) by a priori theme

A priori themes	Emergent subthemes (and % of survey respondents mentioning)
Why researchers should share results	1) Ownership and reciprocity at the participant level (45.7%) 2) Benefits to research/science (31.1%) 3) Closure (26.2%) 4) Benefits to participants/community/public (16.9%) 5) Ethical obligation (15.4%) 6) Reciprocity at the societal level (9.0%) 7) Participants not otherwise receiving results (3.0%)
Why researchers should not share results	1) Health literacy/participants’ comprehension (17.0%) 2) Participants did not ask for results (16.3%) 3) Inconclusive or incomplete results (15.6%) 4) Participant harm (13.6%) 5) Bias or harm research (8.2%) 6) Therapeutic misconception (4.8%) 7) Participant privacy (2.7%) 8) Published results are enough (2.7%) 9) Low participant involvement (2.0%)
Barriers researchers encounter	1) Logistical barriers (68.9%) 2) Researcher barriers (38.1%) 3) Systems barriers (30.8%) 4) Financial barriers (23.0%) 5) Regulatory barriers (10.9%)

Note. Percentages are based on the number of respondents who responded to items relevant to each a priori theme; respondents could have responses coded under more than one emergent subtheme per a priori theme

#### Why researchers should share results

The 267 respondents who responded affirmatively to the question about whether results of research studies should always be shared with participants were asked to write a few sentences to explain why they believe results should be shared with participants. Seven themes emerged within the a priori theme of ‘Why researchers should share results’, as follows: (1) Ownership and reciprocity at the participant level, (2) Benefits to research/science, (3) Closure, (4) Benefits to participants/community/public, (5) Ethical obligation, (6) Reciprocity at the societal level, and (7) Participants not otherwise receiving results.

#### Ownership and reciprocity at the participant level

Of the 267 respondents who believed results should always be shared, 45.7% reported that research participants have given time and/or information (including their bodies, specimens, opinions, etc.), and therefore maintain a degree of ownership of the research results. Respondents described how returning results is an appropriate way for researchers to reciprocate the contribution participants have provided. One respondent captured a frequently recurring claim that “*data really belongs to the participants*” (ID #584). Other respondents stated, “*The participants are the ones who give their time, blood, or access. They deserve first knowledge*” (ID #561) and “*Participants donate their time, their resources, their bodies, and their health records... It is their right to have access to those results in aggregate and our responsibility to share them*” (ID #526).

#### Benefits to research/science

Of the 267 respondents who believed results should always be shared, 31.1% indicated that returning results can benefit research or science by increasing participation, building trust between researchers and participants, and/or helping researchers interpret or improve study findings. Respondents stated that returning results to participants “*promotes long-term public engagement in research*” (ID #73) and “*educates the participants about medical science* [and] *increases likelihood of additional participation*” (ID #578).

#### Closure

Of the 267 respondents who believed results should always be shared, 26.2% described how sharing research results provides closure to study participants or indicates that their participation was valued and important to the researcher and the research process. One respondent summarised: “*Results move science forward and providing results of trials to participants is a way to acknowledge the important role that each participant plays in the scientific process*” (ID #629). Another respondent explained: “*They should see what their participation led to*” (ID #646).

#### Benefits to participants/community/public

Of the 267 respondents who believed results should always be shared, 16.9% mentioned benefits to individual participants, the larger community or the general public. One respondent noted, “*Many patients are curious with regard to the results. They participate often because they want to improve their health or others’ health*” (ID# 96). Another respondent stated, “*Overall the benefit of the project to ‘the world’ as well as to the individual participant should be made available wherever possible*” (ID# 662).

#### Ethical obligation

Of the 267 respondents who believed results should always be shared, 15.4% mentioned ethical considerations as a rationale for why researchers should share results. Respondents discussed returning results to study participants as an ethical responsibility, and the words ‘ethics’ and ‘ethical’ recurred across responses. Respondents stated that “*it is an ethical obligation* [to share results with participants]” (ID# 73). Other respondents elaborated that “*on an ethical level, it is just and fair to provide results to participants*” (ID# 611) and “*I am ethically bound to disseminate information from research studies that I conduct*” (ID# 582).

#### Reciprocity at the societal level

Of the 267 respondents who believed results should always be shared, 9.0% discussed returning results as an appropriate mechanism for researchers to provide reciprocity to the larger public or demonstrate public transparency of research. Respondents explained “*I think we have a duty to share the results… Science should be made public*” (ID #601) and “*Public funding comes with responsibility to determine results and improve healthcare. This includes communicating with the public about study results*” (ID #410).

#### Participants not otherwise receiving results

Of the 267 respondents who believed results should always be shared, 3.0% mentioned the concern that participants do not access the venues in which results are most often reported (e.g. ClinicalTrials.gov or peer-reviewed academic publications); for that reason, participants may not receive results unless researchers share results with them. Respondents stated “*participants might not otherwise learn about the study’s findings*” (ID #586) as a reason they felt obligated to provide results. Others acknowledged that research participants “*would not typically have access to or routinely read the journals in which studies are published*” (ID #503).

#### Why researchers should not share results

The 147 respondents who responded ‘no’ or with uncertainty to the question about whether results of research studies should always be shared with participants were asked to write a few sentences to explain their reservations about always sharing results with participants. Nine themes emerged within the a priori theme of ‘Why researchers should not share results’, as follows: (1) health literacy/participants’ comprehension, (2) participants did not ask for results, (3) inconclusive or incomplete results, (4) participant harm, (5) bias or harm research, (6) therapeutic misconception, (7) participant privacy, (8) published results are enough, and (9) low participant involvement.

#### Health literacy/participants’ comprehension

Of the 147 respondents who did not believe (or were uncertain) that results should always be shared, 17.0% expressed concerns related to participants’ health literacy or general literacy, which could constrain participants’ comprehension of results and/or the research context. Respondents indicated that the “*typical patient would not be able to interpret*” (ID #460) and “*the subjects do not have the scientific basis to understand and might well be caused to be anxious over the findings*” (ID #107).

#### Participants did not ask for results

Of the 147 respondents who did not believe (or were uncertain) that results should always be shared, 16.3% described how results are not always shared because participants do not ask for them or researchers assume participants do not want them. Respondents noted, “*It is not clear to me that all patients actually desire to know the results of the studies*” (ID #322) and “*very few of them* [study participants] *EVER asked for any update*” (ID #25). One respondent indicated, “*They don’t care. Just adds regulatory burden*” (ID #265).

#### Inconclusive or incomplete results

Of the 147 respondents who did not believe (or were uncertain) that results should always be shared, 15.6% cited the inconclusive or incomplete nature of results as a rationale for not sharing them. Respondents discussed how “*some studies give incomplete results or answers to questions being posed. It may be confusing to participants to hear about such results*” (ID #637). Another respondent elaborated, “*it would be inappropriate to release the results of the study if I myself cannot explain the significance of the findings yet*” (ID# 118).

#### Participant harm

Of the 147 respondents who did not believe (or were uncertain) that results should always be shared, 13.6% discussed the idea that study results should not always be shared because researchers did not want to cause physical or emotional harm to participants. Respondents stated, “*It is possible that study results may have adverse consequences for participants if not presented in an appropriate manner*” (ID #652) and “*sometimes the outcomes* [could] *create anxiety in patients*” (ID #569).

#### Bias or harm research

Of the 147 respondents who did not believe (or were uncertain) that results should always be shared, 8.2% indicated that research results should not be shared if they could bias or otherwise diminish the validity or viability of ongoing or future research. Respondents expressed concern that “*results may bias future studies involving these subjects*” (ID #637). Another respondent noted that “*the expense involved in* [sharing results appropriately] *is, in all likelihood, very high and would degrade the ability to support the research mission given the current funding environment*” (ID #10).

#### Therapeutic misconception

Of the 147 respondents who did not believe (or were uncertain) that results should always be shared, 4.8% discussed that study results should not always be shared because participants may not understand that the primary purpose of research is to produce generalisable knowledge and not necessarily to benefit participants in a particular study [35]. Respondents expressed concerns that research participants “*may not understand that these data are to be used for research purposes and are not the same as personal health data they receive from their physicians*” (ID #202). Other respondents elaborated, “*The research conclusions are made on population level, and should not be interpreted as a cause of disease on individual level*” (ID #661) and “*informing the subjects of the results does not provide information that they can act on*” (ID# 605).

#### Participant privacy

Of the 147 respondents who did not believe (or were uncertain) that results should always be shared, 2.7% mentioned privacy concerns. These respondents indicated that study results should not always be shared because results sharing could violate participants’ privacy. Respondents stated that “*some find being contacted after a study is completed intrusive*” (ID #52). “*For example, if receiving the results risked violating participants’ confidentiality (e.g. if receiving a copy of results from a study related to terminated pregnancy inadvertently disclosed that they had terminated a pregnancy), this might not be appropriate*” (ID #119).

#### Published results are enough

Of the 147 respondents who did not believe (or were uncertain) that results should always be shared, 2.7% expressed the belief that publication of peer-reviewed articles that present study findings fulfils researchers’ obligation to share results and that no additional dissemination to participants is required. For example, respondents said “*if the results of the study are available to the public through publication, and participants have access, the results should not have to be shared*” (ID #296). One respondent described how “*when participants request to see the results, I will send them the publication or meeting abstract with aggregated results*” (ID# 671).

#### Low participant involvement

Of the 147 respondents who did not believe (or were uncertain) that results should always be shared, 2.0% indicated that it is not necessary to share results with participants of studies in which participation required only limited involvement. Respondents stated “*some studies’ results are so far removed from the participant’s engagement (e.g. registry studies that then report aggregate results) that they are not meaningful to the subjects*” (ID #633). One respondent noted that “*the studies I have done are brief surveys, and I frankly don’t think that the participants care about the results*” (ID #371).

#### Barriers researchers encounter

The third a priori theme incorporated responses from 357 respondents who responded to three open-ended questions asking them to describe (1) why they did not share results with participants from specific past studies for which they were PI or Co-I, (2) what factors contribute to the difference between the consistent finding that participants generally express a preference to receive results but the equally consistent finding that there is a low probability that participants report having received results, and (3) specific barriers that discouraged them from sharing results with participants. From coding these responses, five themes emerged within the a priori code of ‘Barriers researchers encounter’ as follows: (1) logistical barriers, (2) researcher barriers, (3) systems barriers, (4) financial barriers, and (5) regulatory barriers. The following is an overview of the barriers most frequently reported by respondents.

#### Logistical barriers

Of the 357 respondents who responded to survey items related to barriers, 68.9% described logistical barriers to results sharing. Several respondents indicated that they “*did not have a system in place to share results, or staff to set up such a system*” (ID #625). Respondents discussed difficulty keeping track of participants and discussed a lack of methods to follow-up with participants to return results stating that they “*did not have contact info for the participants*” (ID #673) or a “*substantial portion of participants’ contact information is no longer accurate*” (ID #88).

#### Researcher barriers

Of the 357 respondents who responded to survey items related to barriers, 38.1% cited barriers related to researchers’ awareness and knowledge regarding returning results to participants. Researchers disclosed a lack of attention to the issue, with several respondents making statements such as, “*I don’t think it occurred to me to share the results with the participants*” (ID #669) and “*honestly, I just didn’t think of it*” (ID #462). Others discussed the lack of knowledge and experience with how to share results with study participants, “*I wasn’t sure how to share it, what to share. Share the publication? Share a memorandum of a summary of results?*” (ID #539). Respondents stated that “*writing results for a journal article differs from written results for participants. Writing these results accordingly takes more time and effort*” (ID #88). Specifically, respondents discussed the “*inability to communicate in plain talk*” (ID #693), which would be necessary for sharing results with study participants.

#### Systems barriers

Of the 357 respondents who responded to survey items related to barriers, 30.8% reported a lack of incentive from universities or funding agencies for sharing results with participants. Several respondents pointed out that returning results to participants did not count towards their promotion or tenure and that focusing time and effort on returning results to participants would take time from other activities that were rewarded within the academic system. One respondent wrote, “*I was on the tenure track at that point in my career. My university did not value the time lost to other activities that returning results would require*” (ID #561). Other respondents noted a lack of “*motivation or incentive*” stating that “*journal articles take priority; these documents completed work for funding agencies...funding agencies seldom (never?) require documentation of results for participants*” (ID #88).

#### Financial barriers

Of the 357 respondents who responded to survey items related to barriers, 23.0% described the lack of financial resources required to share study results with participants. Respondents indicated that returning results would be costly, stating that it would be “*too expensive to do mailings or develop an email listserv*” (ID #589). Respondents also mentioned the lack of dissemination funding provided through research grants and contracts, “*there is no extra funding by the sponsor to do dissemination*” (ID #631).

#### Regulatory barriers

Of the 357 respondents who responded to survey items related to barriers, 10.9% discussed concerns about IRB-related regulations. Specifically, respondents shared they worried their IRBs would not permit them to share results with participants if the protocol and/or consent did not specifically include information about returning results to participants. Several respondents described “*IRB concerns*” (ID #388). Respondents expressed a perception that their institutions’ IRB would constrain the return of results to participants, stating that “*an argument must be made to the IRB from the very beginning*” (ID #601).

## Discussion

Our goal in this mixed methods study was to characterise health researchers’ experiences, perceptions and barriers related to sharing de-identified, aggregate information about study findings with participants. Most respondents expressed support for sharing results with participants, and over half of the sample recalled a time when they intended to share results with participants, but never did. The majority of respondents (64.5%) expressed that health research results should always be shared with participants. These findings are consistent with previous smaller studies documenting researchers’ openness toward sharing results with participants [11–16]. This study’s qualitative results suggest that respondents view returning results to participants as a reciprocal exchange for participants’ time and willingness to participate. Echoing themes from indigenous data sovereignty advocates [36] and community-engaged research ethics [37], several respondents acknowledged some degree of research participants’ ownership of their own data. Respondents also mentioned benefits to research, participants and the general public as reasons why researchers should share results.

Consistent with existing smaller studies on sharing research results [11–14, 16], most respondents were able to identify examples of studies for which they did not share results. For most of those studies, respondents had not even considered returning results. Other respondents reported a lack of awareness or knowledge about how to return results to participants. In some instances, researchers failed to communicate results to participants even after telling participants they would receive the results, which could cause participants to view researchers as untrustworthy and undermine willingness to participate further [38–41]. Qualitative findings illuminate several additional reasons why investigators failed to share scientific results with participants. Respondents expressed concerns about the complexity of the results and participant inability to understand or comprehend them. They were also concerned about returning inconclusive or incomplete results to participants, citing the potential for inconclusive data to have unintended, negative consequences for participants or for subsequent research.

This study expands the prior literature by providing new insight into specific reasons researchers do not communicate research results to study participants. Responses to quantitative and qualitative items suggest that failure to share study results may be a function of several barriers that researchers face when attempting to share results with participants. Over one-third of respondents reported financial and logistical issues (e.g. no system in place to share results, inaccurate or no contact information for participants) as barriers to sharing results with participants in at least two-thirds of their studies. Though mentioned with less frequency, respondents also reported systems (e.g. returning results does not help me get tenure) and regulatory issues (e.g. IRB concerns) as barriers to results sharing.

Among the respondents who described their studies where results were not shared with participants, a vast majority (91.7%) disseminated the findings in academic publications and 83.3% presented the results at academic conferences. That researchers overwhelmingly shared study results in publications and at conferences suggests that researchers may place greater value on this type of dissemination or perceive incentives from their academic institutions or funders to promote their results in these formats. If communicating results to participants is a valuable activity, these findings suggest that academic and funding organisations’ policies should incentivise it.

### Limitations

This study has several limitations common to survey studies. For example, it is difficult to determine the extent to which the experiences, perceptions and barriers reported by respondents are representative of those of health researchers in general. Second, due to the number of sites and site-specific email lists implicated in the recruitment process, we were unable to calculate a response rate, as it is unknown how many eligible investigators received invitations. Third, respondents who took time to complete the survey may have been biased in terms of their interest in the topic; however, this challenge occurs frequently in survey research and respondents provided a rich picture of positive and negative attitudes, perceptions, and experiences related to communicating research results to participants.

Fourth, a few researchers’ responses suggested that they did not distinguish between sharing de-identified, aggregate information about study findings with participants versus returning individual research data or incidental findings to specific participants. This confusion speaks to the complexity of the “*distinct ethical and policy issues*” raised by sharing each type of data [42]. While the focus of this study was to provide opportunity for a broad group of health researchers to describe their experiences and concerns related to sharing aggregate study findings with research participants, we would not advocate a one-size-fits-all solution. Detailed discussion of relevant ethical issues is beyond the scope of the present article; however, we point interested readers to the guiding principles of the Multi-Regional Clinical Trials Center of Brigham and Women’s Hospital and Harvard Return of Results workgroup, who noted the importance of (1) offering participants the opportunity to opt-in to receiving concise, plain-language summaries of aggregate findings and (2) incorporating preparation for results sharing throughout institutions’ ethics review processes [43]. That workgroup suggests making participants aware, at the time of study consent, that they will have the option to receive results summaries and to determine with whom, if anyone, they would like these summaries shared.

## Conclusions

This study’s findings make several contributions to the literature on sharing aggregate results with participants. This study’s results replicate existing findings that researchers often fail to share results with participants, despite having positive attitudes and even intentions to do so. This study contributes new, meaningful insights to the literature on scientific communication and results dissemination, documenting barriers to results sharing described by 414 researchers from 44 different academic institutions. The large sample size adds further credibility to these findings.

Taken together, the study’s results reinforce the idea that dissemination efforts require planning early in the study design process [44]. Researchers are open to sharing results with participants, but they perceive significant barriers to sharing; future research in this area should address the extent to which early planning could improve the likelihood that researchers who believe results should be shared are able to do so. Among respondents who expressed reluctance about sharing results with participants, the most common concerns related to participants’ health literacy or ability to comprehend research findings. To address these concerns, academic research institutions with CTSAs should take a leading role. Specifically, academic research institutions should investigate the outcomes of training researchers to communicate findings clearly to participants. More generally, academic research institutions evaluate the extent to which planning tools and a well-defined process for sharing results could persuade and aid researchers to share results with their participants. Furthermore, institutions should investigate ways to address financial, systems and regulatory barriers, and should incentivise researchers through promotion and tenure policies.

## Abbreviations

CBPR: community-based participatory research
Co-I: co-investigator
CTSA: Clinical and Translational Science Award
FTE: full-time equivalent
IRB: Institutional Review Board
PI: principal investigators
PRC: prevention research centers
